# Development of a Real-Time Recombinase-Aided Amplification Method for the Rapid Detection of *Streptococcus equi* subsp. *equi*

**DOI:** 10.3390/microorganisms12040777

**Published:** 2024-04-11

**Authors:** Haoyu Zu, Rongkuan Sun, Jiaxin Li, Xing Guo, Min Wang, Wei Guo, Xiaojun Wang

**Affiliations:** 1 State Key Laboratory for Animal Disease Control and Prevention, Harbin Veterinary Research Institute, Chinese Academy of Agricultural Sciences, Harbin 150069, Chinawangmin202403@163.com (M.W.); 2 College of Veterinary Medicine, Nanjing Agricultural University, Nanjing 210095, China; 3 Institute of Western Agriculture, The Chinese Academy of Agricultural Sciences, Changji 831100, China

**Keywords:** *Streptococcus equi* subspecies *equi*, real-time RAA, strangles

## Abstract

*Streptococcus equi* subspecies *equi* (*S. equi*) is the causative pathogen of strangles in horses, donkeys, and other equine animals. Strangles has spread globally and causes significant losses to the horse industry. In response to the urgent need for effective disease control, this study introduces a novel nucleic acid diagnostic method known as a real-time recombinase-assisted amplification (RAA) assay, developed based on the *eqbE* gene, for the rapid detection of *S. equi* nucleic acid. The real-time RAA method employs specifically designed probes and primers targeting the *eqbE* gene, enhancing the overall specificity and sensitivity of the detection. After efficiency optimization, this real-time RAA method can detect 10 or more copies of nucleic acid within 20 min. The method demonstrates high specificity for *S. equi* and does not cross-react with other clinically relevant pathogens. Real-time RAA diagnostic performance was evaluated using 98 nasal swab samples collected from horses and compared with the real-time PCR detection method. Results revealed that 64 and 65 samples tested positive for *S. equi* using real-time RAA and real-time PCR, respectively. The overall agreement between the two assays was 96.94% (95/98), with a kappa value of 0.931 (*p* < 0.001). Further linear regression analysis indicated a significant correlation in the detection results between the two methods (R^2^ = 0.9012, *p* < 0.0001), suggesting that the real-time RAA assay exhibits a detection performance comparable to that of real-time PCR. In conclusion, the real-time RAA assay developed here serves as a highly specific and reliable diagnostic tool for the detection of *S. equi* in equine samples, offering a potential alternative to real-time PCR methods. In conclusion, the real-time RAA nucleic acid diagnostic method, based on the *eqbE* gene, offers rapid and accurate diagnosis of *S. equi*, with the added advantage of minimal equipment requirements, thus contributing to the efficient detection of strangles in horses.

## 1. Introduction

*Streptococcus equi* subspecies *equi* (*S. equi*), commonly known as the causative agent of equine strangles, is a widespread pathogen that can cause severe upper respiratory tract infections in horses [1,2,3,4,5]. This bacterium is widely distributed in the natural environment and is characterized by clinical manifestations such as fever, and submandibular lymph node enlargement and rupture. In severe cases, it can lead to a fatal outcome resembling strangulation, hence the name “equine strangles” [2,6,7,8]. 

It is worth noting that sporadic cases have been reported where veterinarians or individuals in close contact with infected horses have also contracted the disease [9,10]. Since its discovery, *S. equi* has been rapidly disseminated worldwide [4,11,12,13,14,15,16,17,18], acquiring notable antibiotic resistance [19], and therefore has significant implications for human biosecurity [20]. Carriers of this pathogen may remain asymptomatic, resulting in an undetectable disease state [21,22]. These carriers can transmit the bacteria, harbored within the pharyngeal sac, to other equines through their surroundings, including gear such as tack and grooming equipment, as well as water and fodder [23]. This mode of transmission contributes to widespread strangles epidemics, which have substantial welfare and economic consequences on a global scale. Hence, it has become crucial to employ rapid, accurate, and efficient diagnostic techniques for the detection of equine strangles, as these play a pivotal role in its management, containment, and consequent mitigation of substantial economic losses.

Conventional approaches such as drug sensitivity tests and bacterial culture methods have inherent limitations [24,25]. Time constraints during specimen collection, and extended timelines for sample processing and culture, pose challenges and might yield erroneous results. Furthermore, the phenotypic resemblance of other subspecies of *Streptococcus equi* further complicates accurate identification.

To address these limitations, alternative diagnostic methods have been developed. Polymerase chain reaction (PCR) assays, which offer rapid and highly sensitive detection of *S. equi* DNA in clinical samples, have gained prominence [26,27]. PCR-based techniques provide results within a few hours, enabling timely diagnosis and appropriate management strategies. Additionally, real-time PCR assays allow for quantification of bacterial load, facilitating disease severity assessment and treatment monitoring [28,29,30,31,32].

However, this methodology has limitations as PCR requires intricate procedures, expertise, and costly instrumentation, potentially limiting its application in general organizations, non-specialist labs, and among farmers. Although past diagnostic assays for *S. equi* have played a vital role, they also have their limitations. Therefore, the development of a new assay with higher sensitivity and specificity for the prevention and control of *S. equi* is urgently required.

The recombinase-assisted amplification (RAA) assay is a reliable and innovative diagnostic technique. It utilizes three essential enzymes: recombinase, strand-switching DNA polymerase (for amplification and elongation), and DNA polymerase single-stranded DNA-binding proteins. Amplification products can be detected by adding specific fluorescent probes [33,34]. This method offers a significant advantage as it does not require specialized PCR thermocycling equipment and can be completed in 30 min at a consistently low temperature (37–42 °C). The technique is becoming increasingly important in the discovery of bacteria and viruses and has been extensively applied in research and disease management, showing potential for in situ detection [35,36].

Specifically, our aim is to develop a straightforward, sensitive, and effective diagnostic approach for the detection of *S. equi*. This will involve combining the design and screening of specific probes and primers with the RAA technique and comparing it with the real-time PCR method, which has shown strong diagnostic potential. Our primary focus is to deliver a reliable and efficient diagnostic technique that can be easily implemented in veterinary settings but that ensures the accurate detection of *S. equi*.

## 2. Materials and Methods

### 2.1. Strains of Pathogens, Samples, and Extraction of DNA

*S. equi* (HLJ2018), equine influenza virus (H3 subtype), equine anemia virus, equine herpesvirus type 1 and type 4, equine arteritis virus, and *Escherichia coli* were cultured from clinical samples and maintained at the laboratory of the Equine Infectious Diseases and Lentivirus Research Innovation Team, Harbin Veterinary Research Institute, Chinese Academy of Agricultural Sciences. Nasal samples were collected from suspected sick horses at various horse farms in Heilongjiang Province, China. Genomic DNA or RNA was extracted from the preservative liquid of the collected swabs using the TIANamp Bacteria Genomic DNA Extraction Kit Ver.3.0 (TIANGEN Biotechnology) following the manufacturer’s instructions.

### 2.2. Plasmid

The pUC57-*S.equi*-*eqbE* plasmid was constructed by cloning a partial-length *eqbE* gene (positions 1,226,841 to 1,227,157 of the Se4047 genome) obtained from strain HLJ2018 into the pUC57 vector, using a standard protocol. The resulting plasmid, pUC57-*S.equi*-*eqbE*, was then extracted and purified using a plasmid kit (TianGen Biotech Co., Ltd., Beijing, China) following the manufacturer’s instructions. The purity and concentration of the purified plasmid were assessed using a NanoDrop OneC Spectrophotometer (ThermoFisher, Waltham, MA, USA).

### 2.3. Real-Time RAA Primer and Probe

**Figure 1:**
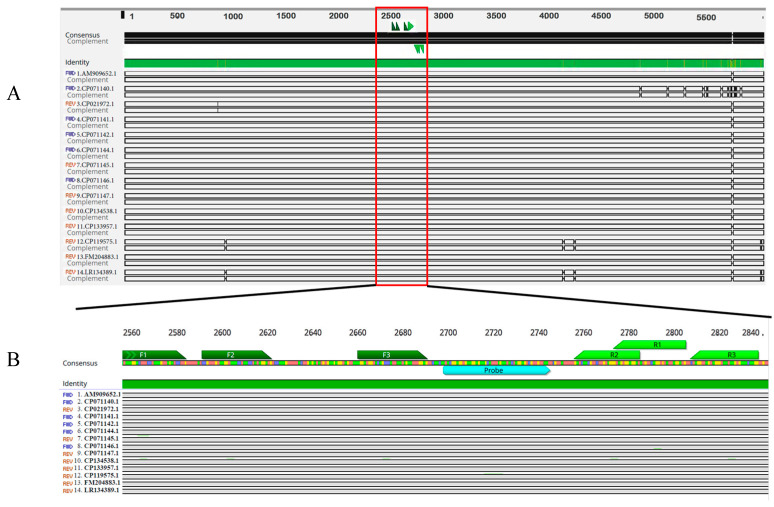
Design of primers and probes for our *eqbE* real-time RAA. (**A**) Schematic representation of conserved regions of the *eqbE* gene as determined with multiple sequence alignment. The 14 full-length *eqbE* sequences from different strains were aligned using Geneious Prime software 9.0.2. The conserved region was selected as the final target for real-time RAA detection and is marked with a red box. The positions of the three designed primer pairs are also shown in (**B**), with the forward primer shown in dark green and the reverse primer in light green. Meanwhile, three forward primers and three reverse primers surrounding the 1,226,874 to 1,227,127 region of the Se4047 (Genbank IDFM204883.1) genome probe were designed and synthesized. These primers were used in a primer screening assay to select the best performing primer pair for the real-time RAA assay (Figure 1B) (Table 1), ensuring optimal amplification fluorescence signal.

To ensure the specificity and sensitivity of the method, the conserved region within *eqbE* (approximately 300 bp, positions 1,226,874 to 1,227,127 of the Se4047 genome) was chosen as the target candidate for the real-time RAA assay (Figure 1A). The selection of the *eqbE* gene was based on its exclusive presence in *S. equi*, which allows for distinguishing between this and other subspecies or external bacteria.

According to the amplification guidelines, the probe for the real-time RAA technique should typically fall in the middle of the sequence and be between 46 and 52 bp in length. The 5′ end of the THF site should generally be at least 30 bp, and the 3′ end at least 15 bp. The fluorophore and quencher group can only be labeled on thymidine (T), and the interval between the fluorophore and quencher group should be 1–5 bp. A longer interval would result in a higher background signal, lower signal-to-noise ratio, and lower quenching efficiency. In this study, the probe (p1,226,969 to 1,227,017) was designed as an *exo* detector, with two conserved thymine residues (T1,227,047, T1,227,050) labeled with a *FAM-dT* fluorophore residue and a *BHQ1-dT* quencher residue, respectively (Figure 1B).

Our design process resulted in the generation of a series of candidate forward and reverse primers, and we were careful to ensure that the primers fell within the range of 30 to 35 base pairs. Primer Premier 5.0 software (Premier Biosoft International, Palo Alto, CA, USA) was used to facilitate accurate primer design.

### 2.4. Real-Time RAA Assay

The real-time RAA Nucleic Acid Amplification Kit (Fluorescent Method) Instruction Manual (Jiangsu Qitian Gene biotechnology Co., Ltd., Wuxi, Jiangsu, China) was followed for the real-time RAA assay. The reaction mixture consisted of 25 μL buffer VI, real-time RAA master mix, 2.1 μL of each primer pair (10 μM), 0.6 μL of the probe (10 μM), and nuclease-free water to reach a final volume of 45 μL. The reaction mixture was carefully closed, vortexed briefly, and centrifuged. Then, 5 μL of magnesium acetate I was added to the tube cap, followed by the nucleic acid sample. The reaction mixture was immediately loaded into an instrument designated MA-688 (MOLARRAY, Suzhou, China) and incubated at 39 °C for 30 min with cycling every half minute. Real-time fluorescence signals were monitored, and samples showing exponential amplification curves higher than that of the negative control within 30 min were considered positive. The negative control sample, which utilized nuclease-free water as its template, underwent the same processing steps as the test sample to monitor and eliminate any potential contamination or false positive results. A flat amplification curve close to the baseline in the negative control is a prerequisite for experiment validity.

### 2.5. Real-Time PCR Assay

Previously reported *eqbE* [26] gene-based real-time PCR assays were performed using an MA-688 real-time quantitative fluorescence instrument (MOLARRAY, China). The reaction mixture (AceQ Universal U + Probe Master Mix V2) contained 10 µL of 2× AceQ Universal U + Probe Master Mix V2, 1 µL of *eqbE* primer F/R (10 µM), 0.5 µL of probe (10 µM), 5.5 µL of nuclease-free water, and 2 µL of the nucleic acid template. The thermal cycling conditions included an initial incubation of 5 min at 95 °C, followed by 2 min at 37 °C, and then 40 cycles of 95 °C for 10 s and 60 °C for 30 s. Samples that produced a cycle threshold (Ct) value of <36 were considered positive.

### 2.6. Analytical Specificity

To evaluate the specificity of the developed real-time RAA for the detection of genomic *eqbE*, templates were prepared from examples of clinically relevant bacteria and viruses from horses, including equine influenza virus (H3 subtype), equine anemia virus, equine herpesvirus type 1 and type 4, equine arteritis virus, and *Escherichia coli*. Templates were then tested using the real-time RAA under the reaction conditions described above. 

### 2.7. Analytical Sensitivity

The number of pUC57-*S.equi*-*eqbE* plasmid copies contained in each microliter standard was calculated as previously described. Real-time RAA assays were performed using a 10-fold gradient dilution of pUC57-*S.equi*-*eqbE* at a concentration of 10^6^ to 10^0^ copies /µL as the template, with 1µL of template added to each reaction tube.

## 3. Results

### 3.1. Design of the eqbE Real-Time RAA Primers and Probe

To evaluate the sensitivity and specificity of the real-time RAA method, we initially selected the *eqbE* gene from *S. equi* as the target. This gene has been reported exclusively from *S. equi*, and not from other subspecies, making it an appropriate diagnostic target.

To identify the most conserved regions of the *eqbE* gene, we aligned the available *eqbE* sequences (positions 1223642 to 1229713 of the Se4047 genome) from the NCBI database using the MAFFT Alignment program in Geneious Prime (Biomatters Ltd., Auckland, New Zealand). This alignment enabled us to identify a highly conserved region spanning nucleotide positions 1,226,841 to 1,227,157 (GenBank no. FM204883.1) (Figure 1A). This specific region, which is approximately 300 bp in length, was selected as the amplification target for the real-time RAA assay.

We had several considerations when designing the probe. Our aim was to position it at the midpoint of the target sequence, with a length ranging from 46 to 52 bp. We ultimately developed an exo detector probe (p1,226,969 to 1,227,017) as an exo detector, with two conserved thymine residues (T1,227,047, T1,227,050) labeled with an FAM-dT fluorophore residue and a BHQ1-dT quencher residue, respectively (see Figure 1B). These design considerations ensure robust and specific detection of the *eqbE* gene in *S. equi*, enhancing the sensitivity and specificity of the real-time RAA method.

### 3.2. Reaction Optimization for Real-Time RAA Assays

**Figure 2:**
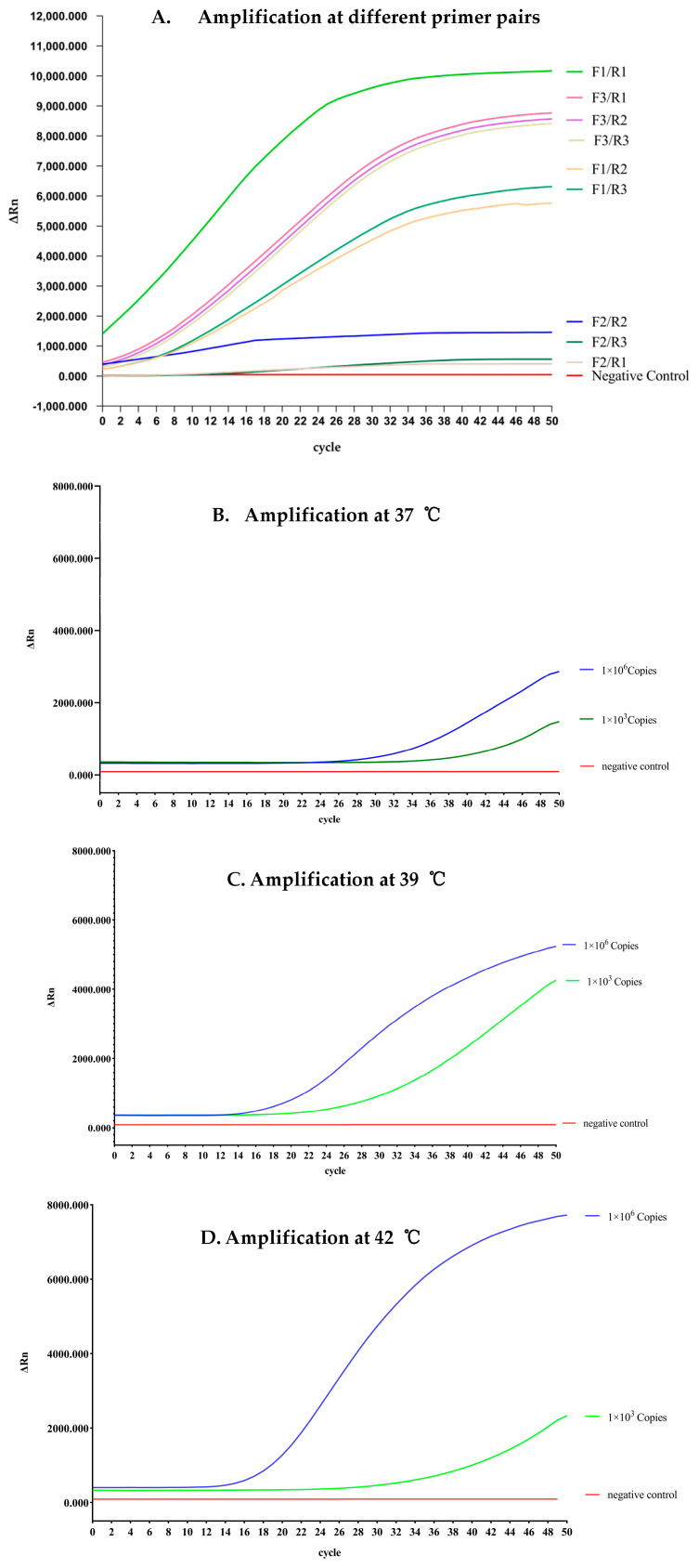
Optimization of real-time RAA reaction for the detection of *eqbE*. (**A**) Representative amplification curves from different primer pairs amplified using real-time RAA are shown. All nine candidate primer pairs (F1/R1-F3/R3) were screened using fixed amplification parameters. The F1/R1 combination produced the highest fluorescence signals in the fewest cycles. (**B**–**D**) Optimization of reaction time and temperature. High and low concentrations of plasmids were each subjected to amplification at temperatures of (**B**) 37 °C, (**C**) 39 °C, and (**D**) 42 °C for 30 min. Representative amplification curves for each temperature are shown.

We then adjusted the reaction temperature and duration to optimize the reaction system of our real-time RAA. Specifically, we employed a standard plasmid, pUC57-*S.equi*-*eqbE*, at 4.173 × 10^8^ copies/μL concentrations representing positive samples. By examining the amplification results, we found that the primer pair F1 and R2 was optimal, as it produced the shortest reaction time and the highest fluorescence signal (Figure 2A).

We conducted amplification experiments using a negative control (water), a strong positive control (1 × 10^6^ copies), and a weak positive control (1 × 10^3^ copies). The resulting products were then assessed using a fluorescence instrument. Each reaction was carried out at constant temperatures of 37 °C, 39 °C, and 42 °C, with a reaction program consisting of 50 cycles (25 min). The amplification curves were then analyzed, leading to the optimized conditions presented in Figure 2B, 2C, and 2D, respectively. 

We observed that at a temperature of 39 °C, the amplification signal for the strongly positive sample exhibited clear visibility, while the amplification signal for the weakly positive sample also appeared rapidly, typically within 30 cycles. The strongly positive sample displayed a robust fluorescence curve, and, similarly, the amplification signal for the weakly positive sample was detected early on, usually around 30 cycles. These findings demonstrate that real-time RAA is capable of efficiently detecting samples containing 1 × 10^3^ copies/μL within a timeframe of 20 min. Based on these results, we have determined 39 °C as the optimal reaction temperature and recommend a reaction time of 20 min. 

### 3.3. Analytical Sensitivity of the Real-Time RAA Assay

**Figure 3:**
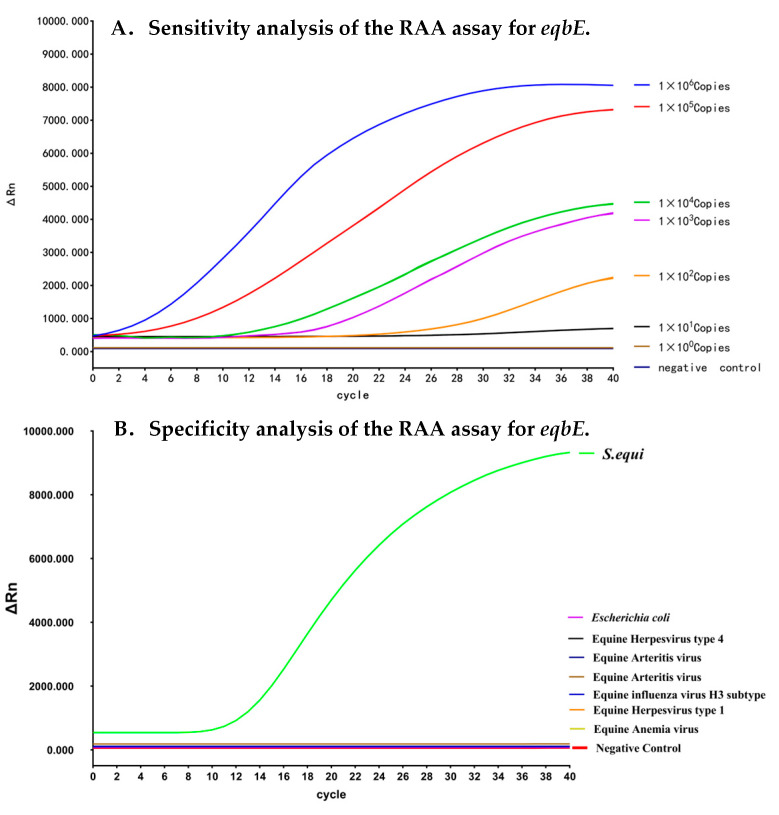
Sensitivity and specificity analyses of the real-time RAA assay for *eqbE*. (**A**) Sensitivity analysis of the real-time RAA assay. Different amplification curves indicate the real-time RAA reaction at different concentrations of plasmids. Serial dilutions of the plasmid were added to the reaction, with dilutions ranging from 10^6^ copies/reaction to 1 copy/reaction. (**B**) Analytical specificity of the real-time RAA assay. The real-time RAA reaction readings, post addition of genomic DNA templates, are illustrated using ion curves of distinct colors. Samples containing the equine influenza virus (H3 subtype), equine anemia virus, equine herpesvirus type 1 and type 4, equine arteritis virus, or *Escherichia coli* all tested negative, while only the sample containing *S. equi* DNA manifested an amplification curve.

We evaluated the sensitivity of the real-time RAA detection method by using 10-fold serial dilutions of positive plasmids as the amplification template. The results are given in Figure 3A. Compared with the negative control, the reaction system containing positive plasmids exhibited clear and reproducible amplification signals. Additionally, higher concentrations of plasmid samples resulted in higher fluorescence signals after amplification. Even when the concentration of the positive plasmid was reduced to 10 copies per reaction, the amplified product fluorescence signal could still be detected. However, when the plasmid concentration was further reduced to one copy per reaction, the amplified product signal disappeared. Therefore, we determined the detection limit of the real-time RAA detection method to be 10 copies per reaction.

### 3.4. Analytical Specificity of the Real-Time RAA Assay

We next analyzed the specificity of the real-time RAA assay. We used nucleic acid samples from other common clinically relevant equine disease pathogens, including equine influenza virus (H3 subtype), equine anemia virus, equine herpesvirus type 1 and type 4, equine arteritis virus, and *Escherichia coli*, for comparison. As depicted in Figure 3B, only the samples from *S. equi* tested positive in our real-time RAA assay, and samples from other diseases all tested negative. This demonstrates that the real-time RAA assay developed in this study exhibits excellent analytical specificity for *eqbE*. 

### 3.5. Diagnostic Performance of Real-Time RAA Using Clinical Nasal Swabs

In order to evaluate the effectiveness of the real-time RAA assay in a clinical setting, a total of 98 nasal swabs were collected from horses on farms with suspected cases of *S. equi*. These samples were subjected to both real-time RAA and real-time PCR testing. The results are summarized in Table 2.

When the real-time RAA assay was used, 66 samples tested positive for *S. equi* and 32 samples tested negative. The real-time PCR test identified 65 individuals as positive for *S. equi*, with 33 individuals testing negative. This yielded an impressive concordance rate of 96.94% (95 out of 98 samples) between the real-time PCR and real-time RAA detection methods.

**Figure 4:**
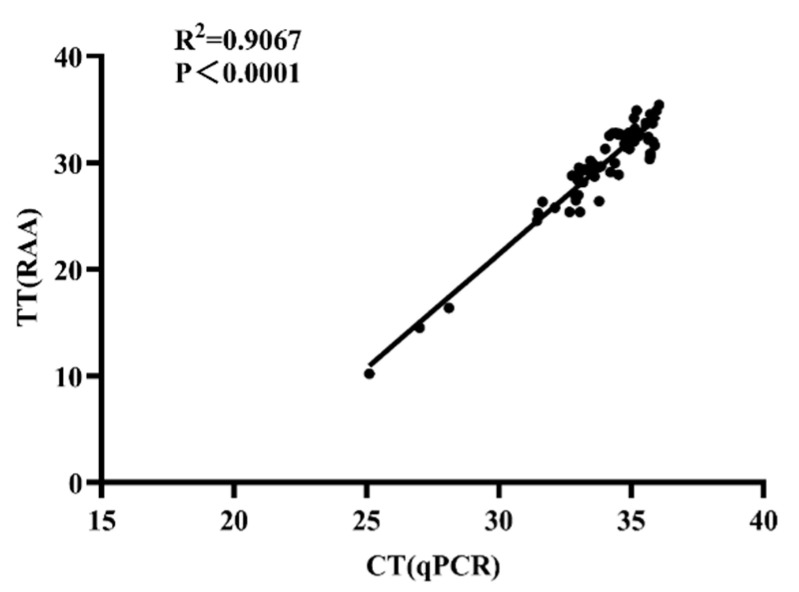
The correlation between real-time RAA and real-time PCR in diagnosing equine strangles in clinical samples. The data are based on 98 clinical swabs from horses on farms with suspected cases of *S. equi*. The Y-axis represents the threshold time (TT) for the real-time RAA and the X-axis shows the cycle threshold (Ct) for the real-time PCR. A linear regression analysis was carried out using GraphPad Prism software 8.0. The results of the two tests showed a strong positive correlation (R^2^ = 0.9012, *p* < 0.0001).

Additional analysis using linear regression and Pearson correlation demonstrated a significant correlation between the real-time PCR and real-time RAA results, as reflected by an R^2^ value of 0.9067 (Figure 4) and a kappa value of 0.931. The real-time RAA assay is therefore a reliable tool for the detection of *S. equi* in the field.

The real-time RAA assay demonstrated excellent sensitivity and specificity, with a concordance rate of 96.94% compared with real-time PCR. This indicates that the real-time RAA assay is highly reliable in identifying the presence or absence of *S. equi* in clinical samples. The significant correlation and high kappa value (0.931) between the real-time RAA and real-time PCR results further reinforces its accuracy as a diagnostic tool in the detection of *S. equi* in clinical nasal swabs. Collectively, these findings highlight the potential of the real-time RAA assay to be employed in clinical settings for the simple, rapid, and accurate diagnosis of strangles, facilitating effective management and control of the disease in equine populations.

## 4. Discussion

Strangles, a prevalent equine disease worldwide, has caused substantial economic losses in the horse industry. Existing diagnostic methods for strangles have limitations in accuracy, speed, and cost-effectiveness. Traditional diagnostic approaches relying on symptom observation and laboratory cultures often lead to delayed diagnosis, susceptibility to interference from other illnesses, and reduced accuracy. Additionally, these methods tend to be time-consuming, delaying timely intervention, and exacerbating the severity of the disease. Moreover, their reliance on complex laboratory procedures and specialized skills restricts their application in the field or in resource-limited environments. The high cost of some laboratory techniques further limits their widespread use, particularly in areas with limited resources. There is therefore an urgent need for a new, simple, fast, accurate, and cost-effective diagnostic method for strangles to improve early detection and timely intervention. This study aimed to address these challenges by developing a novel diagnostic approach based on the design of primers and probes using real-time RAA technology.

**Figure 5:**
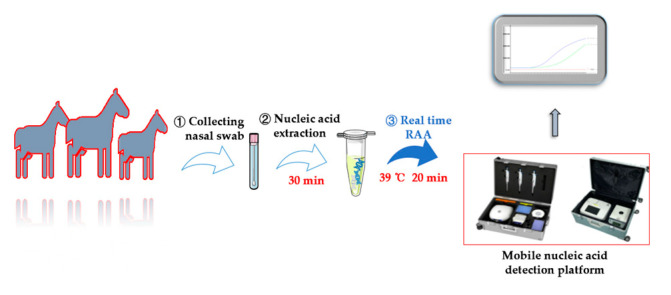
Flowchart of real-time RAA detection. The process begins with the collection of a nasal swab, followed by extracting nucleic acid from the swab’s liquid (approximately 30 min). Subsequently, the reaction solution for real-time RAA is prepared, and nucleic acids from negative, positive, and test samples are added, respectively. Finally, the real-time RAA reaction is conducted on the portable instrument (20 min), and the results can be directly displayed on the screen of the portable detection instrument.

Real-time RAA, an emerging nucleic acid detection method, has shown significant potential compared with traditional PCR technology in disease diagnosis. One of the major advantages of real-time RAA is the minimal equipment required, making it suitable for on-site field applications. In this study, we used real-time RAA technology and designed primers and probes to overcome the limitations of previous diagnostic methods. Following established guidelines, we ensured that the lengths of our primer pairs were 30–35 bases longer than conventional PCR primers. We then identified the optimal primer pairs and probes and further optimized reaction time and temperature. Our real-time RAA detection system achieved high sensitivity (10 copies per sample) and exhibited excellent specificity to *S. equi*. To validate the reliability of this method, we compared its diagnostic performance with that of conventional real-time PCR. The results demonstrated high consistency (95/98) between the two methods, validating the accuracy and reliability of real-time RAA for the detection of *S. equi*. Furthermore, our real-time RAA-based method offers the advantages of short reaction times (20 min), completing at approximately 39 °C under isothermal conditions (Figure 5), while real-time PCR requires longer amplification times. Despite the slightly higher cost associated with real-time RAA, its lower equipment requirements will save considerable capital expenditure, which is of particular importance for the monitoring of strangles in resource-scarce regions. The real-time RAA diagnostic method developed here showed consistently high sensitivity, even at low reaction temperatures. However, these low temperatures potentially elevate the risk of nucleic acid sample contamination. Therefore, rigorous handling of samples and stringent control of reaction conditions are imperative when employing the real-time RAA diagnostic method to ensure accuracy.

In conclusion, our study introduces a novel diagnostic method based on real-time RAA, showcasing flexibility and adaptability, compatible with portable isothermal fluorescence detection devices and existing real-time PCR instruments. This approach facilitates on-site sample testing, reducing reaction time and transportation costs. Our developed real-time RAA diagnostic method holds promise as a potent tool in strangles prevention and control, and should contribute significantly to the health and economics of the equine industry.

## 5. Conclusions

To summarize, this study has successfully devised a real-time RAA assay specifically targeting the *eqbE* gene for the swift detection of equine strangles; in this method, the RAA amplification reaction and signal detection only take 20 min. The entire detection process has been schematically illustrated (Figure 5). The assay showed remarkable sensitivity, with a detection limit of 10 copies/μL, and achieved an impressively high coincidence rate with the real-time PCR technique when applied to clinical samples. Importantly, the real-time RAA assay has the advantages of user-friendliness, efficiency, and compatibility with commonly employed instruments and equipment, making it an invaluable and practical tool for the accurate diagnosis and effective management of strangles.

## Figures and Tables

**Table 1:** Sequences of primers and probes for real-time RAA and real-time PCR.

Name	Sequence (5′-3′)	Location	Reference
*eqbE*-F1	CATCTATTTGGTCAAACCATTTGAATGTACCAAG	1,226,818 to 1,226,852	This study
*eqbE*-F2	CCGAAAGATTGGATTTCCATTCCATATGGTAG	1,226,859 to 1,226,890	This study
*eqbE*-F3	TGGTAGGATCTGCCCTAATTATGTTAAAGGTG	1,226,830 to 1,226,861	This study
*eqbE*-R1	CTACCATTATCTCCAGTTCTATACCACCTCATC	1226, 928 to 1,226,958	This study
*eqbE*-R2	TACCACCTCATCCCATCTTGTTCGAAGTAC	1,226,947 to 1,226,979	This study
*eqbE*-R3	CCAAGAAACTCAATAATCCCATCATTCCATG	1,226,982 to 1,226,912	This study
Probe	TATCGGTGGAGTTGGTGTTGCTAAATGTTA/i6FAMdT//idSp/A/iBHQ1dT/GGTGACGAAGAATTA-3′C3 Spacer	1,226,969 to 1,227,017	This study
*eqbE*-F	ATGTAGCTATGGCAAATGTGGC	1,223,599 to 1,223,620	[26]
*eqbE*-R	AACACCCTTAGGAACACCTG	1,223,689 to 1,223,708	[26]
*eqbE*-probe	FAM-ATTGTTACTATGGCTGAAGGT-BHO1	1,223,966 to 1,223,987	[26]

**Table 2:** Comparison of the performance of *eqbE*-based real-time RAA and real-time PCR for the detection of *Streptococcus equi* subspecies *equi* in clinical samples.

Method		Real-Time PCR			*Kappa*	*p*-Value
		Positive	Negative	Total		
RAA	Positive	64	2	66	0.931	0.0001
	Negative	1	31	32		
Total		65	33	98		

## Data Availability

Data are contained within the article.
